# Assessment of Apolipoprotein(a) Isoform Size Using Phenotypic and Genotypic Methods

**DOI:** 10.3390/ijms241813886

**Published:** 2023-09-09

**Authors:** Federica Fogacci, Valentina Di Micoli, Ashot Avagimyan, Marina Giovannini, Egidio Imbalzano, Arrigo F. G. Cicero

**Affiliations:** 1 Hypertension and Cardiovascular Risk Research Center, Medical and Surgical Sciences Department, Alma Mater Studiorum University of Bologna, 40138 Bologna, Italy; federica.fogacci@studio.unibo.it (F.F.); valentina.dimicoli2@unibo.it (V.D.M.); marina.giovannini3@unibo.it (M.G.); 2 Pathological Anatomy Department, Yerevan State Medical University, Yerevan 0025, Armenia; dr.ashotavagimyan@gmail.com; 3 Department of Clinical and Experimental Medicine, University of Messina, 98125 Messina, Italy; egidio.imbalzano@unime.it; 4 Cardiovascular Medicine Unit, Heart, Thoracic and Vascular Department, IRCCS Azienda Ospedaliero-Universitaria di Bologna, 40100 Bologna, Italy

**Keywords:** lipoprotein(a), Lp(a), apolipoprotein(a), apo(a), KIV 2, LPA gene

## Abstract

Apolipoprotein(a) (apo(a)) is the protein component that defines lipoprotein(a) (Lp(a)) particles and is encoded by the LPA gene. The apo(a) is extremely heterogeneous in size due to the copy number variations in the kringle-IV type 2 (KIV2) domains. In this review, we aim to discuss the role of genetics in establishing Lp(a) as a risk factor for coronary heart disease (CHD) by examining a series of molecular biology techniques aimed at identifying the best strategy for a possible application in clinical research and practice, according to the current gold standard.

## 1. Introduction

During the past decades, the attention on Lipoprotein(a) (Lp(a)) has exponentially grown. Lp(a) is a form of a low-density lipoprotein (LDL) and is an established and genetically determined risk factor for atherosclerosis, coronary artery disease (CAD), stroke, and aortic stenosis [1,2]. Moreover, high plasma levels of Lp(a) nearly double the risk of developing peripheral artery disease [3]. Mendelian studies also associate a high Lp(a) with a mildly though significantly higher risk of developing atrial fibrillation. The pathophysiological link between these two conditions, however, has yet to be clarified [4].

A recent comprehensive meta-analysis of 75 epidemiological studies involving 957,253 participants concluded that the hazard ratios (HRs) and 95% confidence intervals (95%CI) for the top versus the bottom tertiles of Lp(a) levels and the risk of all-cause mortality is 1.09 (95%CI: 1.01 to 1.18) in the general population and 1.18 (95%CI: 1.04 to 1.34) in patients with cardiovascular diseases (CVDs). As expected, the HRs for CVD mortality is 1.33 (95%CI: 1.11 to 1.58) in the general population and 1.25 (95%CI: 1.10 to 1.43) in patients with CVDs, reaching exceptionally high values in patients with type 2 diabetes (2.53 (95%CI: 1.13 to 5.64)). The same analysis highlighted a linear dose–response relationship between Lp(a) and an increased risk of CVD, with a 31% and 15% greater risk of CVD death in the general population and in patients with CVD, respectively, for each 50 mg/dL rise in Lp(a) plasma levels [5].

**Figure 1:**
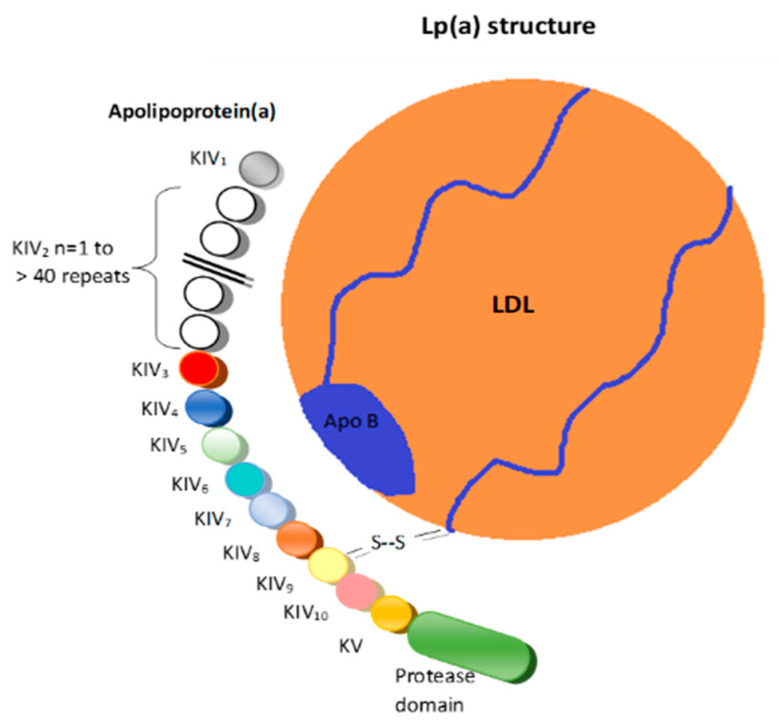
Lipoprotein(a). LDL = Low-density lipoprotein; K = Kringle; Apo B = Apolipoprotein B 100; S-S = Disulfide bridge.

Structurally, Lp(a) is a variant of LDL with an apolipoprotein(a) (apo(a)) that is bound to the apolipoprotein B100 (Figure 1) [6].

These two structures are linked by a disulfide bridge and are assembled in the cell membranes of hepatocytes [7].

The plasma levels of Lp(a) are largely mediated by the LPA gene locus on chromosome 6q22-23, with small or negligible effects resulting from diet [8,9]. The levels of Lp(a) in plasma are determined by the rate of entry of these particles into the circulation (i.e., the production rate) and the efficiency of their removal (i.e., fractional clearance rate) [10].

There is an inverse relationship between the plasma concentrations of Lp(a) and the isoform of apo(a) [11]. The variation in the apo(a) isoform is induced by the different numbers of kringle IV type-2 (KIV-2) repeats in the LPA gene, which leads to variable levels of Lp(a) in the general population [12]. Individuals with fewer kringle repeats have smaller Lp(a) particles but higher serum levels of Lp(a). In addition, the larger the isoforms of apo(a), the greater the accumulation of its intracellular precursor in the endoplasmic reticulum. The short alleles of the KIV-2 copy number variation (CNV) have been shown to be associated with an increased risk of coronary heart disease (CHD) in almost all populations [13].

Recently, the circulating levels of individual apo(a) isoform sizes have been shown to be correlated with the fractionated clearance rate and the production rate of Lp(a). Some studies showed that the isoform size is tightly associated with the Lp(a) production rate (i.e., smaller isoforms with fewer KIV-2 repeats had higher rates of Lp(a) production) [14,15], while other studies showed that the fractionated clearance rate and the production rate of Lp(a) are both affected by the isoform size [16,17].

Lp(a) concentrations in the blood vary by more than a thousand-fold between individuals, ranging from less than 0.1 to more than 300 mg/dL, depending on the size of apo(a) that is encoded by the LPA gene [18]. The plasma concentrations of Lp(a) show considerable variability not only among individuals within a population far exceeding those of other plasma lipoproteins, but they also vary across different human populations [19]. The KIV-2 copy number ranges from 1 to >40, and the CNV of KIV-2 shows a >95% heterozygosity in most populations [20]. Screening patients for elevated Lp(a) is strongly encouraged as an effective tool to identify individuals requiring more aggressive lipid-lowering therapy to reduce the CVD risk [21,22,23,24]. Lp(a) levels above 50 mg/dL are correlated with an increased risk for the development of CVD.

**Figure 2:**
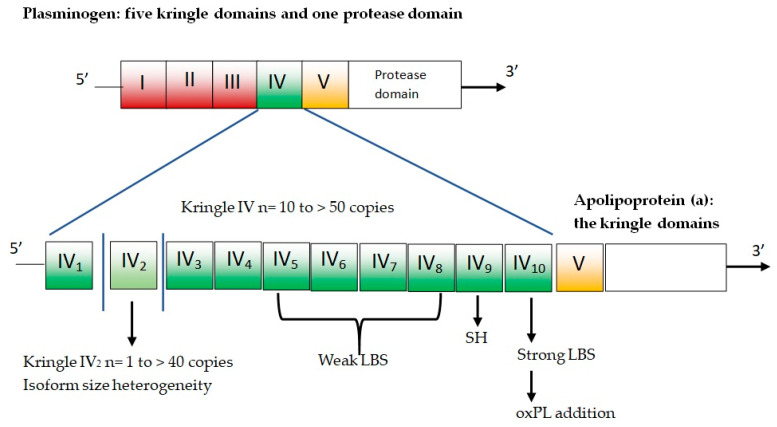
Plasminogen and apolipoprotein(a). LBS = lysine binding sites; oxPL = oxidized phospholipids; SH = free cysteine residue contained in KIV_9_ that mediates covalent coupling to apoB-100 in Lp(a).

Apo(a) is composed of several kringles—which are three-loop structural domains—and a mutated protease domain with no proteolytic activity. The human LPA gene is evolved via duplication from the PLG gene [25]. The PLG gene is characterized by five different kringle domains and one protease domain (Figure 2).

The five plasminogen kringle domains are numbered from 1 to 5 (i.e., KI, KII, KIII, KIV, and KV). In the LPA gene, KI, KII, and KIII are lost by deletion, while KIV sustains mutations and differentiation, resulting in 10 slightly different KIV domains (KIV types 1–10). There is only one copy for each of these domains, except for KIV-2, which can present up to forty copies and represents the variable part of apo(a) [26]. Homozygous individuals express only one isoform, while heterozygotes have two distinct particle types in plasma. The remainder of apo(a) is composed of KV and the mutated protease domain and has a higher number of KIV-2 copies, and larger apo(a) isoform results are associated with lower plasma concentrations of Lp(a) [6].

High levels of Lp(a) may induce a prothrombotic/antifibrinolytic effect because apo(a) resembles both plasminogen and plasmin but does not have fibrinolytic activity. In 20% of children with early arterial ischemic stroke, high levels of Lp(a) were detected [27]. Higher Lp(a) levels have also been detected in patients affected by retinal thrombosis [28]. In particular, the apo(a) KIV-2 copy number variation is associated with a venous thromboembolism risk [29]. The prothrombotic effect of Lp(a) could also contribute to the higher incidence of microvascular diabetic complications, such as nephropathy [30] and feet ulcers [31]. However, it is not yet clear if specific apo(a) isoforms or the Lp(a) mass is directly associated with a higher risk of type 2 diabetes complications. On the other side, high Lp(a) levels are associated with a risk of ischemic stroke [32]. The reason for this observation has also yet to be further explained, but could be related to the individual high structural variability of Lp(a). In addition, high levels of Lp(a) may accelerate atherosclerosis because this particle is rich in cholesterol [33]. The data from pathophysiology, epidemiology and genetics support the causal role of Lp(a) in CVD and aortic valve calcification and stenosis. In fact, meta-analyses of epidemiological studies confirm that its plasma level is a strong predictor of calcium deposition, both in coronary arteries [34] and the aortic valve [35]. 

Small isoforms of Lp(a) are linked to elevated serum Lp(a) levels, and thus, to an increased risk of coronary artery disease. Thus, screening for genetic factors of Lp(a) is expected to have added value and proves to be cost-effective in primary prevention, whereas the monitoring of serum Lp(a) levels could improve the prediction of the clinical risk of atherosclerotic damage in patients with ASCVD [36,37]. 

In this context, the aim of our critical review is to summarize the available genotyping and phenotyping techniques that are potentially able to distinguish subjects at different cardiovascular risks despite having similarly high Lp(a) plasma levels.

## 2. Techniques to Assess the Size of KIV-2 CNV

Several laboratory techniques can be employed to assess the size of the KIV-2 CNV. In this review article, we will explore the pulsed-field gel electrophoresis (PFGE)/Southern blot, the quantitative polymerase chain reaction (qPCR), the fiber fluorescence in situ hybridization (fiber-FISH), and the Western blot (using apo(a) specific antibodies).

### 2.1. Genotyping Techniques

The KIV-2 CNV size polymorphisms can be analyzed to characterize the genetic architecture of Lp(a) [38]. Identifying single nucleotide polymorphisms (SNPs) in the LPA gene has a prognostic significance. The most recent evidence from population studies support the role of some SNIPs (rs6415084 and rs12194138) in predicting future ASCV events independently of the concentration of Lp(a) in the blood [39].

Among the experimental procedures based on deoxyribonucleic acid (DNA) analysis, the largely used PFGE/Southern blot, qPCR, and fiber-FISH will be reviewed thereafter.

#### 2.1.1. Pulsed-Field Gel Electrophoresis (PFGE)/Southern Blot

Variations in the number of LPA KIV2 repeats can be estimated via immunoblotting electrophoresis or the pulsed-field electrophoresis of unamplified genomic DNA. Immunoblotting electrophoresis is used to detect apo(a) protein isoforms [40], whereas the pulsed-field electrophoresis of unamplified genomic DNA is used to detect variations in the LPA gene [41]. These experimental methods are both technically challenging, laborious, and time-consuming. In addition, they require high-quality genetic material and larger amounts of starting DNA than the Southern blot.

APO(a) polymorphisms can be examined via PFGE DNA by means of the use of various restriction enzymes (SwaI, KpnI, KspI, SfiI, and NotI) and an apo(a) kringle-IV-specific probe [42]. According to previous evidence, these enzymes ensure similar results by detecting the same polymorphism in the KIV repeat domain of apo(a) [40]. A PFGE analysis using the KpnI restriction enzyme was found to be able to identify 26 different alleles in unrelated individuals (with sizes ranging from 32 kilobases (kb) to 189 kb, progressively increasing by increments of 5.6 kb corresponding to one KIV unit), with a perfect match between the size of apo(a) DNA phenotypes and the size of apo(a) isoforms in plasma [40]. Unfortunately, PFGE is time-consuming and labor-intensive and can only be performed in reference laboratories with skillful technicians [43]. Furthermore, by measuring the DNA fragments in kb, the molecular weights of the genomic products cannot be derived directly, but alleles are derived from multiples of 5.6 kb. Finally, the post-translational processing of APO(a) cannot be detected.

#### 2.1.2. Quantitative Polymerase Chain Reaction (qPCR)

The qPCR is a faster and more sensitive and reliable assay to detect the number of KIV2 repeats in LPA [44].

One major limitation of the qPCR approach is that it measures the total number of KIV2 repeats instead of the number of KIV2 repeats in each single allele. It follows that qPCR would not always be able to differentiate two different individuals. For instance, an individual who inherited 10 KIV2 repeats from a parent and 20 KIV2 repeats from another parent will exhibit a different biochemical phenotype than another individual who inherited 15 KIV2 repeats from either parent. For the same reason, an individual carrying a null allele would appear to be homozygous for the active allele when using immunoblotting, but qPCR would correctly identify the person as having many KIV2 repeats. Despite these limitations, qPCR is a fast and convenient method to identify the relative total number of KIV2 repeats from genomic DNA samples that have been stored. Moreover, the number of KIV2 repeats in LPA—as assessed using qPCR—has been shown to be correlated with both the size of apo(a) isoforms, as determined via immunoblotting, and the plasma concentration of Lp(a) [45].

#### 2.1.3. Fiber Fluorescence In Situ Hybridization (Fiber-FISH)

The fiber-FISH method is the most precise technique available to determine the size of the KIV-2 CNV as it allows one to count, under a fluorescence microscope, the number of KIV-2 copies on individual alleles [46]. Some unavoidable technical limitations (e.g., the conditions of sample collection, preservation, and storage before analysis) make this method not feasible to handle large sample sizes, although it has historically been used to define standards to be applied in large-scale epidemiologic studies [23,24].

The FISH technique uses high-resolution FITC-labeled 4 kb probes that are intron sequences cloned from KIV-2 repeats [47]. In this way, good linear hybridization signals are obtained on heterozygotes, where there are two different sizes corresponding to different KIV-2 repeat numbers of the respective LPA alleles [48]. A prerequisite for the accurate counting of KIV-2 repeats is to avoid irregular (discontinuous) hybridization signals. For this purpose, to improve the analysis and obtain more reliable results, the conventional agarose plug method for isolating high-molecular-length DNA can be modified. The number of KIV-2 repeats in each allele can then be accurately determined by counting the FISH signals that appear in a “dotted line” rather than a “beadsona string” pattern under a fluorescence microscope [49].

### 2.2. Phenotyping Techniques

Techniques that allow for protein separation such as Western blotting can be used to assess the apo(a) isoform size and associate the plasma concentrations of Lp(a) with specific alleles of LPA.

#### Western Blot Using Antibodies for apo(a) (Immunoblotting)

Sodium dodecyl sulfate (SDS) agarose gel electrophoresis followed by immunoblotting can be used to assess the apo(a) isoform size and associate the relative proportion of the total Lp(a) plasma concentration to a specific LPA allele [49]. This semi-quantitative analysis via densitometry is the most common tool used to assess the specific allele levels of Lp(a) [48]. However, neither the identification of the unexpressed alleles nor the evaluation of samples with a single band in Western blots is feasible without prior knowledge of the actual size of the KIV-2 CNV of both alleles, which could be null or homozygous in size. For this reason, the most reliable assignment between the allele and Lp(a) concentration is ensured by a combination of KIV-2 CNV genotyping and phenotyping by apo(a) [50].

SDS-PAGE (PolyAcrylamide Gel Electrophoresis) with polyacrylamide gel at a low percentage (4%) separates the proteins primarily by mass [51]. However, the high content of carbohydrates in the apo(a) makes it difficult to obtain an accurate molecular weight estimation in SDS-PAGE gels [52]. As shown by Kamboh et al., SDS-agarose is a more efficient method to assess the apo(a) isoform size, also in consideration of the high molecular weight of apo(a) [53]. As an effect, larger proteins are separated more easily in a gel that has a lower percentage of acrylamide because the holes in the web are larger. In addition, the increased migration distance between bands running close together even allows for the detection of very small differences (1 mm resolution limit) between them [54].

## 3. Discussion

Even if the epidemiological and pathophysiological link between the Lp(a) plasma levels and CVD has been suspected some decades ago, the role of high Lp(a) levels as an independent CV risk factor has been underestimated in clinical practice for a long time because of the lack of therapeutic options that are able to significantly reduce them [55]. Diet and physical exercise have nearly no impact on the Lp(a) plasma levels. L-carnitine and coenzyme Q10 are the only nutraceuticals and dietary supplements that mildly improve this parameter, even though their effect is quite irrelevant from clinical and prognostic points of view [56]. Statins have a neutral effect on the Lp(a) plasma levels, balancing the negative effect of Lp(a) on the CV risk when the plasma Lp(a) is less than 50 mg/dL, but not for higher concentrations [57]. Nicotinic acid is the only drug that is able to reduce the Lp(a) by 20-30%, but it is not always well tolerated. Moreover, its use in the long term has never been associated with a significant reduction in CV events [58], even if estimates from genetic studies suggest that Lp(a) decreases might yield proportional reductions in the coronary risk by ≈2% overall and 6% in the top quintile by Lp(a) levels. Additionally, it must be recognized that the effect of nicotinic acid on Lp(a) plasma levels is proportional to the Lp(a) isoform size [59], creating the need for a more refined investigation method of Lp(a) to predict the drug effects. Mipomersen—an antisense oligonucleotide (ASO) directed against apolipoprotein-B 100 (apo B-100) mRNA in the liver—is also able to reduce the Lp(a) levels, but there is serious concern regarding its liver safety [60]. The cholesteryl ester transfer protein (CETP) inhibitors increase the potentially protective high-density lipoprotein (HDL) fraction and reduce the atherogenic non-HDL particles, such as Lp(a) [61]. For instance, according to the findings of a comprehensive meta-analysis of 10 randomized clinical studies (34781 patients overall), anacetrapib significantly lowers the blood concentrations of Lp(a) by a weighted mean difference (WMD) of −13.35 (95%CI: −18.31 to −8.39) [62]. Another CETP inhibitor, evacetrapib, at a dosage of 500 mg as a monotherapy or in combination with statin, has been shown to reduce Lp(a) by 30–40% over a period of 12 weeks [63]. Of course, a CV outcome trial is needed to translate these effects into a reduction in CV events.

Plasma proprotein convertase subtilisin/kexin type 9 (PCSK9) seems to be related to the Lp(a) plasma levels [64]. Hereinafter, the PCSK9 inhibitors and small interfering RNA (siRNA) targeting PCSK9 have been shown to significantly—though mildly—reduce the Lp(a) plasma levels [65,66]. A recent network meta-analysis of 41 randomized controlled trials with 17,601 participants—involving 23 unduplicated interventions—concluded that overall, all the available PCSK9 inhibitors are able to significantly reduce Lp(a) plasma levels (by up to 25.1%), where a biweekly dose of either 140 mg Evolocumab or 150 mg Alirocumab is the best treatment option [67]. Inclisiran was also shown to reduce Lp(a) by an average of 20.9% (95%CI: 25.8% to 15.9%) [68]. However, this effect—though positive—is rarely able to significantly impact the management of patients with high to very high Lp(a) plasma levels. For this reason, to date, in patients with severe and progressive CVD and very high levels of Lp(a), the most effective way to significantly reduce Lp(a) concentrations is apheresis, which is an invasive, expensive, impractical, and not widely available procedure [69]. In a study carried out in patients receiving PCSK9 inhibitors, the size of the apo(a) was shown to be an independent determinant of the treatment response, with each additional kringle domain being associated with a 3% additional reduction in Lp(a) [70]. Currently, emerging lipid-lowering drugs—namely small interfering RNA (siRNA) agents (olpasiran and SLN360) and the second generation ASO pelacarsen—are being developed to interfere with Lp(a) synthesis in the liver by blocking the translation of apo(a) mRNA in apo(a) [71]. These new drugs in development are very promising and overall safe, even if their cost-effectiveness will be carefully evaluated as a part of streamlining the health investments in CV prevention.

According to the most recent evidence, the relative expression of apo(a) isoforms does not change after the Lp(a) levels are lowered using ASO apo(a) treatment [72]. Moreover, the latter results suggest that apo(a) ASO treatment does not preferentially affect one isoform size over the other [10]. However, the knowledge of the available laboratory techniques could steer future research and hopefully help to identify individuals with Lp(a) with a greater atherogenic potential, for whom a reduction in Lp(a) levels with olpasiran or pelacarsen could be more advised and cost-effective.

Notwithstanding that the Lp(a) plasma level is per se an independent predictor of the individual CV risk, Lp(a)-associated CV risk in two individuals with similar Lp(a) levels and apo(a) isoform sizes may differ depending on the relative apo(a) allele expression and/or dominance pattern [73]. In particular, in recent years, two common splice site mutations (G4925A and G4733A with 22% and 38% carrier frequencies, respectively) in the LPA KIV-2 repeat region were shown to have a pronounced Lp(a) decreasing effect, with a concomitant lower risk of coronary artery disease in the carriers [74,75,76]. Although most genetic association studies investigating the potential discordance between the apo(a) isoform size and Lp(a) levels on the artery disease risk provided evidence for a causal role of Lp(a) levels in artery disease independent of the apo(a) isoform size, a Mendelian randomization analysis provided evidence that a variant linked to a smaller apo(a) isoform size, though not to Lp(a) levels, is associated with artery disease [77]. This latter finding makes it even more relevant to balance the different ethnicities in phase 3 clinical trials investigating Lp(a)-lowering drugs, since SNPs and the KIV-2 copy number together explain a greater proportion of the variation in the plasma Lp(a) concentrations in European Caucasians than in Chinese and South Asian individuals [78].

Some cost-effective and practical methods aimed at estimating the Lp(a) mass are not accurate. For instance, the vertical auto profile (VAP) poorly estimates the Lp(a) mass, while it more likely reflects the content of high-density lipoprotein cholesterol (HDL-C) in the overlapping density spectrum of Lp(a) and HDL. For this reason, patients with prior VAP-Lp(a) measurements may have a misclassification of Lp(a)-related risk [79]. Additionally, the assessment of the KIV-2 CNV size could furnish more reliable information on the Lp(a) mass and the related health risks.

Currently, the application of several laboratory techniques can be employed to assess the size of the KIV-2 CNV. However, each method has advantages and disadvantages (Table 1).

The PFGE and fiber-FISH techniques require large amounts of stored, well-preserved, non-fragmented DNA, so that their use is limited by sample collection conditions and technical requirements. Western blotting has been widely used to determine the apo(a) isoform size and associated Lp(a) concentrations, and therefore provides phenotypic information. Compared with PFGE, this method has advantages and drawbacks. Alleles producing apo(a) are recognized, and the amount of Lp(a) contributed by each allele can be determined in heterozygotes with two expressed alleles and exhibiting double-band phenotypes. In these individuals, the CNV genotype can also be directly deduced from the phenotype. However, the frequencies of double-band phenotypes reported in the literature vary considerably, ranging from 30% to 90% [80]. The reasons for this are twofold. Firstly, there are technical limitations. The number of double-band phenotypes depends on the sensitivity of the blotting system and the resolution of the gel system, and both vary among the studies. Moreover, the genetic architecture of the Lp(a)/apo(a) trait in the population is bound to be taken into consideration [80,81]. In fact, populations differ in allele-associated Lp(a) concentrations [82]. Hence, in populations where long alleles are associated with higher concentrations (e.g., populations from Africa), more double-band phenotypes are expected to be detected. Assuming a detection efficiency of 100%, the “true” number of double-band phenotypes to be expected in a population will depend on the number of loss-of-function alleles (null alleles) in that population, which is presently unknown. Furthermore, it is presently difficult to recognize truncated isoforms or splice variants at the protein level without any prior knowledge from molecular analysis. On the other hand, PFGE allows for the determination of the CNV genotype without providing any phenotypic information. In this regard, it should be noted that Simò et al. showed that the correlation of the plasma Lp(a) concentration is stronger with phenotype measures than with genotype measures, even though the application of both methods simultaneously provides the most comprehensive information [83]. It should also be acknowledged that PFGE genotyping needs large amounts of buffy coat, which are not commonly available in population studies [84]. On the contrary, the qPCR technique allows for the rapid assessment of the apo(a) size from DNA, providing an additional genomic variable to assess the genetic determinants of the plasma Lp(a) concentration in epidemiological studies [45,85]. Moreover, compared to PFGE, PCR-based methods are easy to perform and have high levels of specimen typeability and reproducibility.

Recently, a new DRAGEN KIV-2 CN caller—which utilizes short reads—was demonstrated to have high accuracy compared to optical mapping across 166 whole genome sequencing, and can further phase ∼50% of the samples. The authors compared the KIV-2 CN numbers to 24 previously postulated KIV-2-relevant SNVs, finally showing that many are ineffective predictors of KIV-2 copy numbers [86].

A recent study carried out on 1.020 individuals including 252 clinically diagnosed hypercholesterolemia patients from the Familial Hypercholesterolemia (FH) Register Austria showed that the integration of polygenic scores with some LPA gene variants increases the proportion of individuals with a clearly defined disease by nearly 30% compared to standard scores [87]. Moreover, a genome-wide association study (GWAS) identified within the LPA/PLG locus seven SNPs associated with CHD events in individuals undergoing statin treatment. Each copy of the risk allele G at the lead variant, rs10455872, was associated with a 58% increased risk of CHD events, with this association also being present in individuals with LDL-C ≤ 70 mg/dL [88]. All of the previous considerations have to be put in the context of the single country’s public health policies and priorities. Of course, an advanced investigation of the Lp(a) isoforms and genetics make sense, whereas CVD prevention is a national public health priority, quantitative Lp(a) screening is already widely available, and the access to second-level lipid-lowering treatments exists and is supported by the public health system and/or insurances. In the United States, for instance, universal Lp(a) screening in the youth has been estimated to be feasible and cost-effective [89]. In these circumstances, the simultaneous application of methods for both the phenotyping and genotyping aspects [46] in association with the CV risk—especially for the most studied techniques including Western blotting with SDS agarose [90,91] and qPCR [29,92,93,94]—provide more complete information for the personalized management of individuals with an increased CV risk. Of course, identifying individuals with high Lp(a) levels and a higher CV risk could improve the cost-effectiveness of the novel or up-coming Lp(a)-lowering drugs. Additionally, currently, the application of these methods should be limited to the study of specific family clusters and/or of individuals with recurrent CV events without other CV risk factors expect for hyperlipoproteinemia(a). Further research is needed to improve the available phenotyping and genotyping techniques to validate their prognostic value and increase their availability, reducing their cost.

## Figures and Tables

**Table 1:** Main features of the laboratory techniques that can be employed to assess the size of KIV-2 CNV.

Experimental Technique	Level of Information	Investigated Molecule	Outcome	Setting	Main Strengths	Main Limitations
Western Blot (SDS-PAGE agarose)	Phenotype	apo(a) (protein)	Double-band visualization in heterozygotes	Clinical Practice	It detects allele-specific apo(a) levels and the amount of Lp(a) contributed by each allele in heterozygote individuals	Post-translational alterations The number of double-band phenotypes depends on the sensitivity of the blotting system and the resolution of the gel system
PFGE	Genotype	DNA	Displaying the sum of KIV-2 CNV over alleles	Clinical Practice	It ensures a perfect match between the size of apo(a) DNA phenotypes and the size of apo(a) isoforms in plasma	Time-consuming and labor-intensive technique It allows for the determination of the CNV genotype without providing any phenotypic information (does not allow for distinguishing between homozygous and heterozygous individuals) Large amounts of well-preserved non-fragmented DNA needed Operator-dependent variability
qPCR	Genotype	DNA	Displaying the sum of KIV-2 CNV over alleles	Clinical Practice	Time-efficient technique High levels of specimen typeability and reproducibility	It does not ensure the determination of the number of KIV-2 repeats in each allele (does not allow for distinguishing between homozygous and heterozygous individuals)
Fiber-FISH	Genotype	DNA	Microscopic visualization of KIV-2 CNV on both alleles separately	Population Studies	It ensures an accurate determination of the number of KIV-2 repeats in each allele	Large amounts of well-preserved non-fragmented DNA needed

Apo(a) = Apolipoprotein(a); CNV= Copy number variation; DNA = Deoxyribonucleic acid; FISH = Fluorescence in situ hybridization; KIV = Kringle IV; PAGE = Polyacrylamide gel electrophoresis; PFGE = Pulsed-field gel electrophoresis; qPCR = Quantitative polymerase chain reaction; SDS = Sodium dodecyl sulfate.

## Data Availability

Not applicable.
